# A common variant within the HNF1B gene is associated with overall survival of multiple myeloma patients: Results from the IMMEnSE consortium and meta-analysis

**DOI:** 10.18632/oncotarget.10665

**Published:** 2016-07-18

**Authors:** Rafael Ríos-Tamayo, Carmen Belén Lupiañez, Daniele Campa, Thomas Hielscher, Niels Weinhold, Joaquin Martínez-López, Andrés Jerez, Stefano Landi, Krzysztof Jamroziak, Charles Dumontet, Marzena Wątek, Fabienne Lesueur, Rui Manuel Reis, Herlander Marques, Artur Jurczyszyn, Ulla Vogel, Gabriele Buda, Ramón García-Sanz, Enrico Orciuolo, Mario Petrini, Annette J Vangsted, Federica Gemignani, Asta Försti, Hartmut Goldschmidt, Kari Hemminki, Federico Canzian, Manuel Jurado, Juan Sainz

**Affiliations:** ^1^ Genomic Oncology Area, GENYO. Centre for Genomics and Oncological Research: Pfizer/University of Granada/Andalusian Regional Government, Granada, Spain; ^2^ Hematology Department, Virgen de las Nieves University Hospital, Granada, Spain; ^3^ Department of Biology, University of Pisa, Pisa, Italy; ^4^ Division of Biostatistics, German Cancer Research Center (DKFZ), Heidelberg, Germany; ^5^ Myeloma Institute, University of Arkansas for Medical Sciences, Little Rock, Arkansas, USA; ^6^ Department of Internal Medicine V, University of Heidelberg, Heidelberg, Germany; ^7^ Department of Hematology, Hospital Universitario Doce de Octubre, Madrid, Spain; ^8^ Hematology and Medical Oncology Department, University Hospital Morales Meseguer, IMIB, Murcia, Spain; ^9^ Medical University of Lodz, Lodz, Poland; ^10^ INSERM UMR 1052/CNRS 5286, Universite Claude Bernard Lyon I, Lyon, France; ^11^ Hematoloy Clinik, Holy Cross Cancer Center, Kielce, Poland; ^12^ Institute Curie, Paris, France; ^13^ PSL Research University, Paris, France; ^14^ INSERM, U900, Paris, France; ^15^ Mines ParisTech, F-77305 Cedex Fontainebleau, France; ^16^ Life and Health Sciences Research Institute (ICVS), School of Health Sciences, University of Minho, Braga, Portugal and ICVS/3B's – PT Government Associate Laboratory, Braga/Guimarães, Portugal; ^17^ Molecular Oncology Research Center, Barretos Cancer Hospital, Barretos, Brazil; ^18^ Jagiellonian University Medical College, Department of Haematology, Kraków, Poland; ^19^ National Research Centre for the Working Environment, Copenhagen, Denmark; ^20^ UO Hematology, Department of Internal and Experimental Medicine, University of Pisa, Pisa, Italy; ^21^ Haematology Department, University Hospital of Salamanca & IBSAL, Salamanca, Spain; ^22^ Department of Hematology, Rigshospitalet, Copenhagen, Denmark; ^23^ Molecular Genetic Epidemiology, German Cancer Research Center (DKFZ), Heidelberg, Germany; ^24^ National Center of Tumor Diseases, Heidelberg, Germany; ^25^ Genomic Epidemiology Group, German Cancer Research Center (DKFZ), Heidelberg, Germany

**Keywords:** multiple myeloma, diabetes, genetic variants, survival

## Abstract

Diabetogenic single nucleotide polymorphisms (SNPs) have recently been associated with multiple myeloma (MM) risk but their impact on overall survival (OS) of MM patients has not been analysed yet. In order to investigate the impact of 58 GWAS-identified variants for type 2 diabetes (T2D) on OS of patients with MM, we analysed genotyping data of 936 MM patients collected by the International Multiple Myeloma rESEarch (IMMENSE) consortium and an independent set of 700 MM patients recruited by the University Clinic of Heidelberg. A meta-analysis of the cox regression results of the two sets showed that rs7501939 located in the *HNF1B* gene negatively impacted OS (HR_Rec_= 1.44, 95% CI = 1.18–1.76, *P* = 0.0001). The meta-analysis also showed a noteworthy gender-specific association of the *SLC30A8*_rs13266634_ SNP with OS. The presence of each additional copy of the minor allele at rs13266634 was associated with poor OS in men whereas no association was seen in women (HR_Men-Add_ = 1.32, 95% CI 1.13–1.54, *P* = 0.0003). In conclusion, these data suggest that the HNF1B_rs7501939_ SNP confers poor OS in patients with MM and that a SNP in SLC30A8 affect OS in men.

## INTRODUCTION

Multiple myeloma is an incurable and heterogeneous plasma cell neoplasm that affects about 6.3 per 100.000 people per year worldwide (i.e., 25.850 new cases only in 2015) and represents 1.6% of all cancers and 2% of all cancer deaths [1]. In spite of the widespread use of proteasome inhibitors and immunomodulatory drugs, which has dramatically improved the life expectancy of MM patients over the last few decades [2, 3], MM survival still remains poor with a 5-year survival of 46.6% (SEER Cancer Statistics Review, http://seer.cancer.gov/csr/1975_2012/).

Epidemiological and observational studies have consistently identified several factors that affect MM patient survival such as age at diagnosis [4, 5], stage at diagnosis (coded by either the Durie-Salmon staging system (DSS) [6] or the International Staging System (ISS)) [7], Eastern Cooperative Oncology Group (ECOG) performance status [8], renal failure [9, 10], high plasma cell proliferative rate [11, 12], high lactate deshydrogenase (LDH) levels [13] and chromosomal abnormalities [14–18]. Increasing evidences point towards a positive correlation of pre-existing type 2 diabetes (T2D) with MM risk [19] but also with the appearance of severe clinical complications [20–23] and patient survival [24, 25]. In this regard, Chiu et al. (2006) reported that high level of postload glucose was associated with increased risk of mortality in hematological malignancies [24] whereas Chou et al. (2012) reported that MM patients with pre-existing T2D have 50% higher all-cause mortality compared with non-diabetic patients [25]. These observations might be explained, at least in part, by the stimulatory effects of T2D-associated hyperglycaemia, insulin resistance and resulting hyperinsulinemia on MM cell growth [26, 27] but also by the deregulation of tumour-suppressor genes linked to T2D (such as CDKN2A-2B, KCNQ1, HNF1B) [28–30] that might lead to uncontrolled cell proliferation, cell differentiation and disease progression and, consequently, to shorter survival periods. In support of this notion, CDKN2A-2B genes have been found to be frequently hypermethylated in MM [31–33] whereas loss of expression of KCNQ1 has been associated with poor overall survival in cancer patients [34]. Furthermore, emerging evidences also suggest that the activation of certain T2D-related genes (such as NOTCH2) may induce MM cell migration from the infiltrated site to different bone marrow districts [35] and promote osteoclast formation [36], which is a process intimately related to proliferation and long-term survival of MM cells [37].

Although germline variants may influence the susceptibility of MM [38–43] and survival [39, 44–46], the knowledge regarding the role of diabetogenic variants in modulating the risk of MM and survival remains scarce. We have recently reported that diabetogenic variants influence MM risk [47] and recent genome-wide association studies (GWAS) have also suggested the involvement of genetic variants within the *MTHFD1L*, *AKAP12* and *FOPNL* loci in determining MM patient survival [39, 44, 45] but also an indirect implication of diabetogenic genes such as *TCF7L2* [45]. Johnson *et al.* (2016) reported in their GWAS a strong association of rs12374648, which maps to a binding site for the transcription factor *TCF7L2*, with MM overall survival (OS) and proposed a functional mechanism of this variant to modulate the synthesis of purines and the regulation of cell cycle [45].

Based on these findings, we explored for the first time the relationship between diabetogenic variants and OS of MM patients in a study developed in the context of the International Multiple Myeloma rESEarch (IMMENSE) consortium. We attempted to confirm our findings by analysing GWAS data on an independent set of German MM patients (Heidelberg cohort) [45].

## RESULTS

The demographic and clinical characteristics of the MM patients included in the IMMENSE (*n* = 939) and Heidelberg (*n* = 700) cohorts are listed in Table 1. The median age at diagnosis was similar in both populations (59.73 ± 10.08 vs. 55.85 ± 8.33) but the male/female ratio was higher in the Heidelberg cohort (1.36 vs. 1.06). Durie-Salmon stage was available for IMMENSE and Heidelberg cohorts and included patients at stages I, II and III (11.83%, 23.54% and 64.63% vs. 0.8%, 12.4% and 86.8%, respectively).

**Table 1:** Clinical characteristics of IMMEnSE and Heidelberg cohorts

IMMENSE population			
Country of origin	MM patients (*n* = 936)		
	Gender M/F (Total)	Mean Age (± STD)	Median Age (Range)
*Italy*	69/69 (138)	61.31 ± 9.47	51.0 (35–86)
*Poland*	145/163 (308)	62.48 ± 10.50	52.0 (34–86)
*Spain*	49/55 (104)	62.44 ± 11.45	66.0 (22–88)
*France*	42/33 (75)	55.80 ± 9.04	41.0 (34–75)
*Portugal*	14/22 (36)	65.22 ± 9.54	35.0 (45–80)
*Denmark*	163/112 (275)	55.18 ± 7.32	51.0 (29–69)
***Demographic variables***			
*Age (years, average ± SD)*		59.73 ± 10.08	
*Sex ratio (male/female)*		1.06 (482/454)	
***Overall survival (months)***		99.69 [92.98,106.39]	
*Number of deaths*		323	
*Median follow-up time (months)*		100 (52–111)	
Disease stage (Durie-Salmon)^*^			
*Stage I*		93 (11.83)	
*Stage II*		185 (23.54)	
*Stage III*		508 (64.63)	

Heidelberg population			
	MM patients (*n* = 700)		
Country of origin	Gender M/F (Total)	Mean Age (± STD)	Median Age (Range)
*Germany (HD3 trial)*	56/42 (98)	55.42 ± 7.47	56.5 (38–65)
*Germany (HD4 trial)*	170/121 (291)	55.02 ± 7.30	57.0 (27–65)
*Germany (non-trial)*	178/133 (311)	56.76 ± 9.37	57.2 (24–73)
***Demographic variables***			
*Age (years, average ± SD)*		55.85 ± 8.33	
*Sex ratio (male/female)*		1.36 (404/296)	
***Overall survival (months)^∂^***		91.2 [85.5,105]	
*Number of deaths*		326	
*Median follow-up time (months)*		84.3 (80–88)	
***Disease stage (Durie-Salmon)***			
*Stage I*		5 (0.8)	
*Stage II*		82 (12.4)	
*Stage III*		575 (86.8)	

Abbreviations: IMMENSE, International Multiple Myeloma rESEarch; MM, Multiple Myeloma; SD, standard deviation.

* Durie-Salmon data was not available for 150 MM patients.

∂ Median overall survival after diagnosis (IMMENSE) or the 1st autotransplant (Heidelberg cohort) (KM estimators).

**Figure 1:**
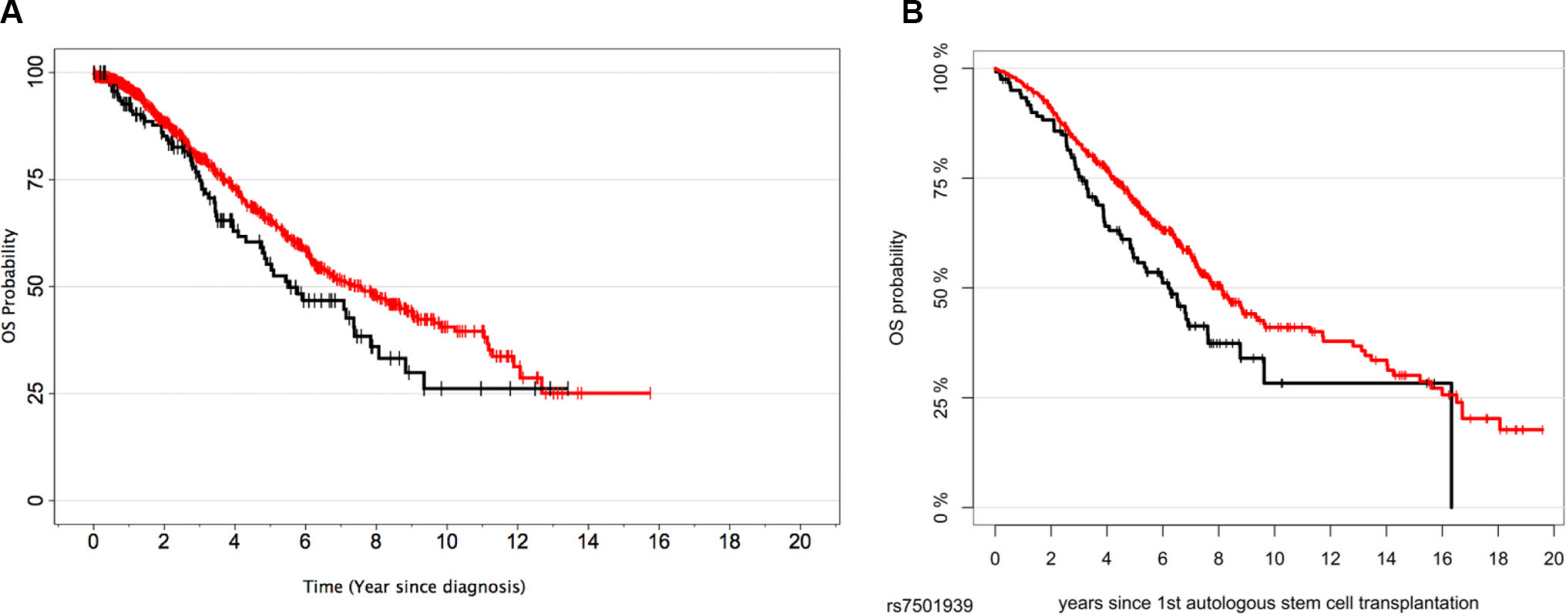
Kaplan-Meier plots for the *HNF1B*_rs7501939_ SNP in the IMMENSE (A) and Heidelberg (B) populations

All SNPs tested showed genotype frequencies consistent with the HWE (*P* > 0.001) and the observed allele frequency for all selected SNPs was in accordance with Hapmap data. When we evaluated the effect of selected polymorphisms on MM OS in the IMMENSE population, we found that 6 SNPs showed a noteworthy association with OS. The most relevant effect was observed for the *HNF1B*_rs7501939_ SNP that was associated with poor OS when recessive and log-additive models of inheritance were assumed (HR_REC_ = 1.49, 95% CI 1.11–2.00, *P* = 0.008 and HR_ADD_ = 1.34 95% CI 1.13–1.59, *P* = 0.001, respectively; Table 2). Patients harbouring the non-diabetogenic *HNF1B*_rs7501939T/T_ genotype showed a median survival time (MST) significantly shorter than those carrying the C allele (MST_T/T_ = 81.91 months vs. MST_C/C+C/T_ = 101.42 months; Figure 1A). This result was confirmed with the 700 MM patients recruited from the University Clinic of Heidelberg (HR_REC_ = 1.40, 95% CI 1.06–1.84 and MST_T/T_ = 74.4 months vs. MST_C/C+C/T_ = 97.2 months; Table 3 and Figure 1B). The result of the meta-analysis for this SNP remained significant after correction for multiple testing (HR_Meta-Rec_ = 1.44, 95% CI 1.18–1.76, *P* = 0.0001, I^2^ = 0.0%, *P*_Het_ = 0.74; Table 3). According to publicly available eQTL data for human peripheral blood mononuclear cells, the risk allele (T) was associated with higher *HNF1B* mRNA expression levels (Z score = 3.31, *P* = 9.23·10^–4^ and FDR = 0.23). However, we could not validate this finding using eQTL data on plasma cells from 665 German MM patients (*P* = 0.60; Supplementary Table S1). Nonetheless, according to Haploreg and ENCODE annotation data, the rs7501939 SNP resides near of a poised promoter in many cell lines including a lymphoblastoid and human stem cell lines (GM12878 and H1-HSCs) that might be rapidly activated upon specific stimuli. In addition, this SNP was predicted to change binding motifs for 2 regulatory transcription factors (CEBPB and p300) and mapped among enhancer histone marks in primary naïve and memory forms of cytotoxic T cells (CD_8_^+^) and helper T cells (CD_4_^+^) from peripheral blood.

**Table 2:** Association of T2D–related variants and overall survival (OS) of MM patients

Variant_dbSNP	Gene	OVERALL (*N* = 936)		MEN (*N* = 482)		WOMEN (*N*= 454)		
		OR (95% CI)^a^	*P_value_*	OR (95% CI)^b^	*P_value_*	OR (95% CI)^b^	*P*_value_	*P*_Interaction_
rs2641348	*ADAM30*	0.94 (0.69–1.28)	0.69	0.99 (0.67–1.48)	0.97	0.87 (0.53–1.41)	0.56	0.70
rs4607103^†^	*ADAMTS9*	1.23 (0.76–2.00)	0.40	0.83 (0.42–1.63)	0.58	**2.53 (1.26–5.07)**	**0.009**	0.024
rs11708067	*ADCY5*	0.87 (0.68–1.11)	0.25	1.02 (0.75–1.39)	0.89	**0.63 (0.42–0.95)**	**0.028**	0.08
rs10885122	*ADRA2A*	1.03 (0.79–1.33)	0.83	1.01 (0.72–1.40)	0.97	1.07 (0.71–1.62)	0.73	0.89
rs1552224	*ARAPI, CENTD2*	0.88 (0.68–1.15)	0.35	0.90 (0.64–1.26)	0.55	0.83 (0.54–1.25)	0.37	0.77
rs10490072	*BCL11A*	1.05 (0.83–1.32)	0.70	1.22 (0.90–1.65)	0.19	0.85 (0.59–1.23)	0.39	0.11
rs12779790	*CDC123, CAMK1D*	0.86 (0.67–1.11)	0.24	1.06 (0.77–1.46)	0.72	**0.64 (0.43–0.97)**	**0.035**	0.06
rs7754840	*CDKAL1*	1.14 (0.90–1.44)	0.27	1.22 (0.90–1.65)	0.20	1.06 (0.74–1.53)	0.75	0.52
rs564398^†^	*CDKN2A–2B*	**0.64 (0.42–0.98)**	**0.042**	0.62 (0.36–1.07)	0.084	0.71 (0.34–1.47)	0.36	0.88
rs10811661	*CDKN2A–2B*	0.93 (0.72–1.19)	0.55	0.93 (0.67–1.30)	0.68	0.92 (0.63–1.35)	0.67	0.92
rs2383208	*CDKN2A–2B*	0.94 (0.73–1.21)	0.64	0.92 (0.66–1.29)	0.64	0.95 (0.65–1.41)	0.81	0.87
rs4240702	*COL5A1*	0.88 (0.68–1.15)	0.35	0.85 (0.60–1.21)	0.36	0.95 (0.63–1.44)	0.82	0.65
rs11605924	*CRY2*	0.91 (0.70–1.18)	0.47	0.81 (0.58–1.14)	0.23	1.09 (0.71–1.69)	0.68	0.29
rs1153188	*DCD*	1.18 (0.94–1.48)	0.16	1.25 (0.92–1.69)	0.15	1.09 (0.76–1.57)	0.63	0.58
rs1113132	*EXT2*	1.02 (0.81–1.28)	0.86	0.96 (0.71–1.30)	0.79	1.08 (0.75–1.56)	0.66	0.57
rs174550	*FADS1*	1.00 (0.79–1.26)	1.00	0.97 (0.72–1.30)	0.82	1.05 (0.73–1.51)	0.80	0.71
rs11071657	*FAM148B*	0.86 (0.68–1.09)	0.22	0.96 (0.70–1.32)	0.81	0.77 (0.53–1.12)	0.17	0.44
rs17044137	*FLJ39370*	1.06 (0.84–1.33)	0.64	1.07 (0.79–1.44)	0.67	1.03 (0.71–1.50)	0.87	0.92
rs8050136	*FTO*	0.91 (0.70–1.18)	0.46	0.81 (0.58–1.13)	0.21	1.10 (0.72–1.67)	0.66	0.23
rs560887^†^	*G6PC2*	1.11 (0.74–1.66)	0.61	0.78 (0.44–1.39)	0.40	1.76 (1.00–3.09)	0.050	0.045
rs1799884	*GCK*	0.99 (0.76–1.28)	0.92	1.10 (0.80–1.52)	0.57	0.81 (0.52–1.26)	0.36	0.39
rs1260326^†^	*GCKR*	1.36 (1.01–1.82)	0.043	1.24 (0.84–1.83)	0.28	1.53 (0.97–2.42)	0.068	0.47
rs1111875	*HHEX*	1.00 (0.78–1.27)	0.97	0.92 (0.67–1.26)	0.61	1.19 (0.80–1.77)	0.38	0.33
rs7957197	*HNF1A (TCF1)*	1.17 (0.92–1.48)	0.20	1.18 (0.87–1.59)	0.30	1.16 (0.80–1.70)	0.43	0.89
rs7501939^†^	*HNF1B (TCF2)*	**1.49 (1.11–2.00)**	**0.008**	**1.49 (1.03–2.17)**	**0.036**	1.49 (0.91–2.45)	0.11	0.99
rs35767	*IGF1*	0.87 (0.67–1.12)	0.27	0.78 (0.56–1.10)	0.16	1.00 (0.68–1.48)	1.00	0.40
rs4402960	*IGF2BP2*	0.90 (0.71–1.13)	0.36	0.78 (0.58–1.06)	0.11	1.10 (0.76–1.60)	0.60	0.16
rs20541^†^	*IL13*	1.42 (0.89–2.27)	0.14	0.93 (0.45–1.90)	0.84	**2.38 (1.24–4.57)**	**0.009**	0.07
rs2943641	*IRS1*	1.13 (0.89–1.43)	0.33	1.01 (0.75–1.38)	0.93	1.38 (0.94–2.02)	0.10	0.30
rs864745	*JAZF1*	0.90 (0.71–1.15)	0.40	1.01 (0.73–1.39)	0.98	0.75 (0.52–1.09)	0.14	0.22
rs5215	*KCNJ11*	1.09 (0.86–1.37)	0.49	**1.39 (1.02–1.90)**	**0.038**	0.79 (0.55–1.13)	0.20	**0.022**
rs5219	*KCNJ11*	1.11 (0.87–1.43)	0.39	1.37 (0.98–1.91)	0.063	0.88 (0.60–1.28)	0.50	0.12
rs2237897	*KCNQ1*	1.25 (0.88–1.77)	0.21	1.28 (0.81–2.01)	0.28	1.22 (0.71–2.10)	0.48	0.83
rs2074196	*KCNQ1*	1.57 (1.03–2.40)	0.036	**1.83 (1.01–3.32)**	**0.047**	1.42 (0.77–2.59)	0.26	0.48
rs2237892	*KCNQ1*	1.38 (0.97–1.97)	0.070	1.53 (0.97–2.41)	0.065	1.24 (0.70–2.18)	0.46	0.53
rs2237895	*KCNQ1*	1.06 (0.82–1.36)	0.66	1.30 (0.94–1.80)	0.11	0.74 (0.50–1.11)	0.15	0.033
rs231362	*KCNQ1OT1*	1.15 (0.88–1.51)	0.31	1.04 (0.73–1.47)	0.84	1.34 (0.87–2.07)	0.19	0.33
rs1041981	*LTA*	0.86 (0.68–1.09)	0.21	0.76 (0.56–1.04)	0.088	1.04 (0.71–1.51)	0.85	0.25
rs7944584^†^	*MADD*	0.68 (0.46–1.01)	0.058	0.55 (0.32–0.95)	0.031	0.83 (0.46–1.52)	0.55	0.27
rs12970134	*MCR4*	0.89 (0.70–1.12)	0.31	**0.86 (0.64–1.16)**	**0.31**	0.94 (0.65–1.35)	0.74	0.71
rs1387153	*MTNR1B*	0.89 (0.71–1.12)	0.32	1.01 (0.75–1.37)	0.93	0.71 (0.49–1.02)	0.063	0.16
rs10923931	*NOTCH2*	0.98 (0.73–1.31)	0.88	1.05 (0.72–1.53)	0.81	0.88 (0.55–1.41)	0.59	0.58
rs6698181	*PKN2*	1.17 (0.92–1.48)	0.20	1.28 (0.94–1.75)	0.12	1.00 (0.69–1.46)	0.98	0.33
rs1801282	*PPARG*	0.84 (0.65–1.10)	0.21	**0.66 (0.46–0.96)**	**0.030**	1.12 (0.76–1.65)	0.56	0.09
rs8042680^†^	*PRC1*	0.91 (0.64–1.29)	0.60	1.13 (0.74–1.73)	0.56	0.56 (0.29–1.07)	0.081	0.08
rs340874	*PROX1*	1.03 (0.78–1.36)	0.83	0.75 (0.52–1.07)	0.11	**1.60 (1.02–2.50)**	**0.041**	**0.016**
rs7593730	*RBMS1*	0.92 (0.73–1.16)	0.48	0.77 (0.56–1.05)	0.10	1.20 (0.83–1.75)	0.33	0.07
rs1531343	*RPSAP52, HMGA2*	1.11 (0.84–1.46)	0.45	1.40 (1.00–1.97)	0.053	0.76 (0.46–1.23)	0.26	0.07
rs11920090	*SLC2A2*	0.88 (0.67–1.14)	0.34	0.87 (0.62–1.22)	0.41	0.91 (0.59–1.40)	0.67	0.91
rs13266634^∂^	*SLC30A8*	**1.24 (1.05–1.47)**	**0.011**	**1.42 (1.14–1.77)**	**0.002**	1.01 (0.77–1.33)	0.94	0.057
rs7903146	*TCF7L2*	0.83 (0.66–1.05)	0.12	**0.72 (0.53–0.97)**	**0.030**	1.04 (0.72–1.50)	0.82	0.13
rs12255372	*TCF7L2*	0.87 (0.69–1.09)	0.23	**0.73 (0.54–0.98)**	**0.039**	1.14 (0.79–1.65)	0.49	0.08
rs7578597	*THADA*	1.18 (0.88–1.58)	0.27	0.97 (0.67–1.41)	0.88	1.59 (0.99–2.55)	0.054	0.12
rs896854^†^	*TP53INP1*	0.76 (0.58–1.00)	0.050	0.74 (0.52–1.06)	0.10	0.77 (0.50–1.19)	0.24	0.81
rs7961581	*TSPAN8, LGR5*	1.09 (0.87–1.37)	0.47	1.08 (0.80–1.47)	0.62	1.14 (0.79–1.63)	0.49	0.77
rs9472138	*VEGFA*	1.03 (0.82–1.30)	0.80	0.93 (0.69–1.26)	0.63	1.19 (0.83–1.71)	0.33	0.32
rs10010131	*WFS1*	1.00 (0.78–1.28)	0.99	1.15 (0.81–1.61)	0.44	0.85 (0.59–1.23)	0.38	0.28

Abbreviations: SNP, single nucleotide polymorphism; OR, odds ratio; CI, confidence interval; n/s, not specified.

Estimates were adjusted for age, sex, country of origin and Durie–Salmon stage.*P* < 0.05 in bold.

a Estimates calculated according to a dominant model of inheritance.

b Estimates calculated according to a dominant model of inheritance and adjusted for age, region and Durie–Salmon stage.

† Estimates calculated according to a recessive model of inheritance.

∂ Estimates calculated according to an additive model of inheritance.

**Table 3:** Meta–analysis for the association of T2D–related variants and overall survival (OS) of MM patients

		IMMENSE (*N* = 936)		GWAS (*N* = 700)		META-ANALYSIS (*N* = 1636)	
Variant_dbSNP	Gene	OR (95% CI)^a^	*P_value_*	HR (95% CI)^b^	*P_value_*	HR (95% CI)^c^	*P_value_*
rs2641348	*ADAM30*	0.94 (0.69–1.28)	0.69	1.12 (0.86–1.45)	0.41	1.04 (0.85–1.27)	0.69
rs4607103^†^	*ADAMTS9*	1.23 (0.76–2.00)	0.40	0.99 (0.63–1.55)^*^	0.98	1.10 (0.79–1.52)^*^	0.59
rs11708067	*ADCY5*	0.87 (0.68–1.11)	0.25	0.89 (0.71–1.11)^*^	0.31	0.88 (0.75–1.04)^*^	0.13
rs10885122	*ADRA2A*	1.03 (0.79–1.33)	0.83	0.85 (0.65–1.12)	0.24	0.94 (0.78–1.13)	0.52
rs1552224	*ARAPI, CENTD2*	0.88 (0.68–1.15)	0.35	1.12 (0.89–1.42)	0.34	1.00 (0.79–1.27)	1.00
rs10490072	*BCL11A*	1.05 (0.83–1.32)	0.70	1.30 (1.04–1.62)^*^	**0.019**	1.17 (0.95–1.44)^*^	0.14
rs12779790	*CDC123, CAMK1D*	0.86 (0.67–1.11)	0.24	ND	ND	ND	ND
rs7754840	*CDKAL1*	1.14 (0.90–1.44)	0.27	1.15 (0.92–1.43)	0.22	1.14 (0.98–1.35)	0.10
rs564398^†^	*CDKN2A–2B*	**0.64 (0.42–0.98)**	**0.042**	1.17 (0.88–1.55)	0.29	0.88 (0.49–1.59)	0.68
rs10811661	*CDKN2A–2B*	0.93 (0.72–1.19)	0.55	ND	ND	ND	ND
rs2383208	*CDKN2A–2B*	0.94 (0.73–1.21)	0.64	ND	ND	ND	ND
rs4240702	*COL5A1*	0.88 (0.68–1.15)	0.35	1.12 (0.87–1.44)	0.39	1.00 (0.79–1.26)	0.97
rs11605924	*CRY2*	0.91 (0.70–1.18)	0.47	1.05 (0.82–1.35)^*^	0.70	0.98 (0.82–1.18)^*^	0.83
rs1153188	*DCD*	1.18 (0.94–1.48)	0.16	0.85 (0.68–1.07)^*^	0.16	1.00 (0.73–1.38)^*^	0.99
rs1113132	*EXT2*	1.02 (0.81–1.28)	0.86	1.01 (0.81–1.26)	0.93	1.02 (0.87–1.19)	0.86
rs174550	*FADS1*	1.00 (0.79–1.26)	1.00	1.10 (0.88–1.37)	0.39	1.05 (0.90–1.24)	0.54
rs11071657	*FAM148B*	0.86 (0.68–1.09)	0.22	1.01 (0.81–1.27)	0.91	0.94 (0.80–1.10)	0.42
rs17044137	*FLJ39370*	1.06 (0.84–1.33)	0.64	1.04 (0.83–1.31)^*^	0.71	1.05 (0.89–1.23)^*^	0.56
rs8050136	*FTO*	0.91 (0.70–1.18)	0.46	1.02 (0.81–1.29)	0.84	0.97 (0.82–1.15)	0.73
rs560887^†^	*G6PC2*	1.11 (0.74–1.66)	0.61	0.91 (0.61–1.35)	0.63	1.01 (0.77–1.33)	0.94
rs1799884	*GCK*	0.99 (0.76–1.28)	0.92	1.04 (0.83–1.31)^*^	0.74	1.02 (0.86–1.21)^*^	0.84
rs1260326^†^	*GCKR*	**1.36 (1.01–1.82)**	**0.043**	1.10 (0.83–1.47)	0.51	1.22 (0.99–1.50)	0.061
rs1111875	*HHEX*	1.00 (0.78–1.27)	0.97	1.00 (0.79–1.25)^*^	0.97	1.00 (0.85–1.18)^*^	1.00
rs7957197	*HNF1A (TCF1)*	1.17 (0.92–1.48)	0.20	1.07 (0.85–1.35)^*^	0.56	1.12 (0.95–1.32)^*^	0.19
rs7501939^†^	*HNF1B (TCF2)*	**1.49 (1.11–2.00)**	**0.008**	**1.40 (1.06–1.84)**	**0.016**	**1.44 (1.18–1.76)**	**0.0001**
rs35767	*IGF1*	0.87 (0.67–1.12)	0.27	1.08 (0.85–1.37)	0.53	0.98 (0.79–1.20)	0.81
rs4402960	*IGF2BP2*	0.90 (0.71–1.13)	0.36	0.90 (0.72–1.12)	0.34	0.90 (0.77–1.06)	0.20
rs20541^†^	*IL13*	1.42 (0.89–2.27)	0.14	0.82 (0.48–1.41)	0.47	1.10 (0.64–1.88)	0.73
rs2943641	*IRS1*	1.13 (0.89–1.43)	0.33	1.08 (0.86–1.36)	0.49	1.10 (0.94–1.30)	0.24
rs864745	*JAZF1*	0.90 (0.71–1.15)	0.40	1.01 (0.79–1.30)^*^	0.94	0.95 (0.80–1.13)^*^	0.58
rs5215	*KCNJ11*	1.09 (0.86–1.37)	0.49	0.98 (0.78–1.23)	0.85	1.03 (0.88–1.22)	0.70
rs5219	*KCNJ11*	1.11 (0.87–1.43)	0.39	0.98 (0.78–1.23)	0.85	1.04 (0.88–1.23)	0.67
rs2237897	*KCNQ1*	1.25 (0.88–1.77)	0.21	ND	ND	ND	ND
rs2074196	*KCNQ1*	**1.57 (1.03–2.40)**	**0.036**	ND	ND	ND	ND
rs2237892	*KCNQ1*	1.38 (0.97–1.97)	0.070	1.09 (0.80–1.49)	0.59	1.21 (0.96–1.53)	0.11
rs2237895	*KCNQ1*	1.06 (0.82–1.36)	0.66	0.94 (0.75–1.19)	0.62	0.99 (0.84–1.18)	0.93
rs231362	*KCNQ1OT1*	1.15 (0.88–1.51)	0.31	1.04 (0.80–1.35)	0.76	1.09 (0.91–1.32)	0.36
rs1041981	*LTA*	0.86 (0.68–1.09)	0.21	0.95 (0.76–1.18)	0.64	0.91 (0.77–1.07)	0.24
rs7944584^†^	*MADD*	0.68 (0.46–1.01)	0.058	0.83 (0.56–1.24)^*^	0.37	0.75 (0.57–0.99)^*^	**0.044**
rs12970134	*MCR4*	0.89 (0.70–1.12)	0.31	1.11 (0.89–1.39)	0.34	1.00 (0.80–1.24)	0.98
rs1387153	*MTNR1B*	0.89 (0.71–1.12)	0.32	1.12 (0.90–1.30)	0.30	1.01 (0.81–1.26)	0.94
rs10923931	*NOTCH2*	0.98 (0.73–1.31)	0.88	1.10 (0.85–1.44)^*^	0.48	1.04 (0.86–1.27)^*^	0.66
rs6698181	*PKN2*	1.17 (0.92–1.48)	0.20	0.97 (0.78–1.21)	0.77	1.06 (0.88–1.27)	0.54
rs1801282	*PPARG*	0.84 (0.65–1.10)	0.21	0.90 (0.70–1.16)^*^	0.41	0.87 (0.73–1.05)^*^	0.14
rs8042680^†^	*PRC1*	0.91 (0.64–1.29)	0.60	1.05 (0.75–1.47)	0.77	0.98 (0.77–1.25)	0.87
rs340874	*PROX1*	1.03 (0.78–1.36)	0.83	0.81 (0.64–1.03)^*^	0.08	0.90 (0.71–1.14)^*^	0.40
rs7593730	*RBMS1*	0.92 (0.73–1.16)	0.48	1.09 (0.87–1.36)^*^	0.44	1.00 (0.85–1.19)^*^	0.96
rs1531343	*RPSAP52, HMGA2*	1.11 (0.84–1.46)	0.45	0.88 (0.66–1.17)^*^	0.39	0.99 (0.79–1.25)^*^	0.94
rs11920090	*SLC2A2*	0.88 (0.67–1.14)	0.34	**0.73 (0.56–0.95)**	**0.022**	**0.80 (0.66–0.97)**	**0.020**
rs13266634^∂^	*SLC30A8*	**1.24 (1.05–1.47)**	**0.011**	**1.20 (1.02–1.41)**	**0.025**	**1.22 (1.09–1.37)**	**0.001**
rs7903146	*TCF7L2*	0.83 (0.66–1.05)	0.12	0.84 (0.67–1.05)	0.12	**0.84 (0.71–0.98)**	**0.028**
rs12255372	*TCF7L2*	0.87 (0.69–1.09)	0.23	0.83 (0.67–1.04)	0.10	0.85 (0.73–1.00)	**0.043**
rs7578597	*THADA*	1.18 (0.88–1.58)	0.27	0.95 (0.73–1.24)	0.72	1.05 (0.85–1.30)	0.66
rs896854^†^	*TP53INP1*	0.76 (0.58–1.00)	0.050	0.97 (0.75–1.25)^*^	0.82	0.86 (0.68–1.10)^*^	0.23
rs7961581	*TSPAN8, LGR5*	1.09 (0.87–1.37)	0.47	0.92 (0.74–1.14)	0.44	1.00 (0.85–1.18)	0.98
rs9472138	*VEGFA*	1.03 (0.82–1.30)	0.80	1.10 (0.88–1.37)	0.39	1.07 (0.91–1.25)	0.43
rs10010131	*WFS1*	1.00 (0.78–1.28)	0.99	1.07 (0.86–1.35)	0.54	1.04 (0.88–1.23)	0.66

Abbreviations: SNP, single nucleotide polymorphism; HR, hazard ratio; CI, confidence interval. ND, not determined.

Estimates were adjusted for age, sex, country of origin and Durie–Salmon stage. *P* < 0.05 in facebold.

a Estimates calculated according to a dominant model of inheritance and adjusted for age, gender, region and Durie–Salmon stage.

b Estimates calculated according to a dominant model of inheritance and adjusted for age, gender and clinical trial.

c Meta–analyses were performed assuming a random effect model.

† Estimates calculated according to a recessive model of inheritance.

∂ Estimates calculated according to an additive model of inheritance.

* Estimates based on imputed genotypes.

We also observed significant associations at *P* < 0.05 for SNPs within *CDKN2A-2B*, *GCKR, KCNQ1* and *SLC30A8* genes with OS in the IMMENSE population. Thus, patients carrying the *KCNQ1*_rs2074196T_ and *SLC30A8*_rs13266634T_ alleles or the *GCKR*_rs1260326T/T_ genotype had an increased risk of death whereas subjects bearing the *CDKN2A-2B*_rs564398C/C_ genotype showed longer OS (Table 2 and Supplementary Tables S2–S4). The association of the *SLC30A8*_rs13266634T_ allele with OS was confirmed in the Heidelberg population and the meta-analysis showed that the presence of each additional copy of the *SLC30A8*_rs13266634T_ allele was associated with poor OS (HR_Meta-Add_ = 1.22, 95% CI 1.09-1.37; Table 3). Although the association of the *SLC2A2*_rs11920090_ SNP with OS was not significant in the IMMENSE population, we observed a significant association of this variant with MM survival in the Heidelberg population that remained significant in the pooled analysis. Patients harbouring the *SLC2A2*_rs11920090T_ allele showed a better survival compared with those carrying the A/A genotype (HR_Meta-Dom_ = 0.80, 95% CI 0.66-0.97; Table 3). The meta-analysis also showed a weak association of the *TCF7L2*_rs7903146T_ allele with better survival that was neither significant in the IMMENSE population nor in the Heidelberg cohort (HR_Meta-Dom_ = 0.84, 95% CI 0.71–0.98). Based on Haploreg data, the missense rs13266634 SNP was predicted to change binding motifs for transcription factors implicated in tumorigenesis (AP1 and PAX5) and mapped on enhancer histone marks in several human embrionic stem cell lines. In addition, this polymorphism affects binding to 5 proteins implicated in cancer development (CCNT2, GATA2, TAL1, KAP1 and CTCF). On the other hand, the rs11920090 and rs7903146 SNPs mapped among enhancer and promoter histone marks in bone marrow- and/or adipose-derived mesenchymal stem cells. In addition, the rs7903146 SNP was predicted to alter the binding site of 7 transcription factors. However, despite the consistency and the potential interest of these findings, none of the associations of the *SLC2A2*, *SLC30A8*, and *TCF7L2* SNPs with OS remained significant after correction for multiple testing and, therefore, require further confirmation. Given the lack of genetic information in the GWAS conducted in the Heidelberg population for SNPs within *MADD and KCNQ1* genes, we imputed genotypes to test whether the preliminary associations observed in the IMMENSE population could be validated. Although there was no imputed data available for the *KCNQ1* SNP, the meta-analysis of IMMENSE data with imputed genotypes of *MADD* variants in the Heidelberg cohort suggested a link between this locus and MM survival (HR_Meta-Rec_ = 0.75, 95% CI 0.57–0.99; Table 3).

Based on the evidences that point toward the existence of gender-associated differences in survival for patients with MM [48], we decided to carry out a gender-stratified analysis. This analysis revealed gender-specific associations for SNPs within or near the *ADAMTS9*, *KCNJ11, PROX1* and *SLC30A8* genes with OS. We found that men carrying the *KCNJ11*_rs5215C_ or *SLC30A8*_rs13266634T_ alleles had poorer OS compared with those harbouring the wild type genotype whereas an opposite but not significant effect was seen in women (*P*_Interaction_= 0.022 and *P*_Interaction_ = 0.057, respectively; Table 2 and Supplementary Table S2–S4). We also observed that women carrying the *PROX1*_rs340874G_ allele or the *ADAMTS9*_rs4607103T/T_ genotype experienced a poorer survival with an opposite but not significant effect in men (*P*_Interaction_ = 0.016 and *P*_Interaction_ = 0.024). In order to confirm these gender-specific associations, we performed a meta-analysis with available GWAS data of the Heidelberg population. Although there was a partial overlapping of SNPs between both populations that limited our ability to validate some potentially interesting gender-associated effects on OS, we could confirm the strong association of the *SLC30A8*_rs13266634_ SNP with OS in men that could not be detected in women (per-allele HR_Men_ = 1.32, 95% CI 1.13-1.54; Supplementary Table S5). This gender-specific association remained significant at the experiment-wide significance threshold. On the other hand, although it was not statistically significant in the analysis of the IMMENSE population, the pooled analysis also showed that men carrying the *BCL11A*_rs10490072C_ allele had a poorer OS compared with those carrying the wild type genotype whereas no effect was seen in women (HR_Men_ = 1.37, 95% CI 1.10-1.70). Finally, we observed in the pooled analysis that women bearing the *PRC1*_rs8042680A_ allele or men carrying the *PROX1*_rs340874G_ allele or the *MADD*_rs7944584T/T_ genotype showed a significantly better OS when compared with those patients carrying the corresponding wild type allele or genotype (HR_Women_ = 0.62, 95% CI 0.39–0.98; HR_Men_ = 0.74, 95% CI 0.59–0.94 and HR_Men_ = 0.59, 95% CI 0.39–0.87; Supplementary Table S5). The regulatory characteristics of the rs10490072, rs8042680 and rs7944584 SNPs were changes in transcription binding motifs for transcription factors involved in tumorigenesis and T- and B-cell malignancies. The rs10490072 changed sites for HNF4B, Pou2f2 and Pou5f1 whereas the rs8042680 altered sites for GR, PAX5 and TAL1. The rs7944584 was found to modify regulatory motifs for AP1, AP4, IRF and KAP1. Finally, the rs340874 mapped among promoter and enhancer histone marks in primary naïve and memory forms of helper T cells (CD_4_^+^) and regulatory T cells from peripheral blood.

## DISCUSSION

Previous population-based studies have demonstrated the impact of GWAS-identified variants for T2D on cancer susceptibility [47, 49–52] and patient survival [53]. However, despite these important research advances, there is still a noticeable lack of information regarding the role of T2D-related variants in modulating patient survival especially in hematological malignancies. In this scenario, we decided to investigate for the first time to our knowledge the relationship between 58 genetic variants associated with T2D identified by GWAS and OS of MM patients.

The analysis of the IMMENSE consortium data revealed a significant association of the intronic *HNF1B*_rs7501939_ SNP with poor OS. We successfully replicated this association in a large and independent population recruited by the University Clinic of Heidelberg. However, although a positive correlation between this variant and eQTL data on PBMCs has been reported [54], we failed to find correlation between the risk allele and *HNF1B* mRNA expression levels on plasma cells from a large cohort of MM patients. This suggested that the effect of this variant on overall survival is not mediated by changes in transcriptional activity of the gene. Nonetheless, given that *HFN1B* contains multiple independent SNPs or haplotypes that have been associated with *HNF1B* mRNA expression [55, 56] and methylation [57] levels but also with the risk of developing several types of cancer [55–57], it seems to be reasonable to consider the possibility that other SNPs within this locus and showing a stronger association with OS could explain better the link between the *HNF1B* and clinical outcome. However, when we analysed imputed common SNPs from the GWAS conducted in the Heidelberg cohort, we could not find any stronger association signals with OS in the region, which suggested that the *HNF1B*_rs7501939_ SNP or perhaps a rare SNP in LD with it might be responsible of the observed effect. Future fine-mapping studies encompassing common but also rare variants within or near the *HNF1B* locus are needed to elucidate whether a rare variant or haplotype might account for the observed effect.

*HNF1B* contains 9 exons and expands over 58 kb on chromosome 17p21 [58, 59]. It encodes for a transcription factor that has been associated with multiple clinical features including early-onset of T2D [56]. In line with this, it has been also suggested that *HNF1B* may induce impaired glucose tolerance and attenuated insulin sensitivity in a miRNA-dependent manner [60], which might lead to an enhanced insulin secretion and the activation of the *IGF1* pathway, an important factor mediating myeloma cell growth, proliferation and cell maturation [27, 59, 61]. Alternatively, it has been postulated that *HNF1B* is able to influence cancer cell survival by promoting the activation of NFkB pathway or through the inhibition of mitochondria-associated apoptotic signals [62]. In support of the tumorogenic effect of HFN1B, it has also been reported that it may act as an oncogene [63] and that the HNF1B gene is amplified in 23% of all cancers and in about 5% of all haematological malignancies (http://broadinstitute.org/tumorscape). On the contrary, it has also been reported that HFN1B may act as tumour suppressor gene [56] and that its expression may largely vary depending on the target tissue. Whereas HNF1B has been found to be overexpressed in ovarian clear cell carcinomas [58] and prostate [64] or endometrial [65] cancers and its silencing induces apoptosis of cancer cells [58], it has been found to be down-regulated in serous epithelial ovarian cancer [57] and colorectal, gastric and pancreatic cancer cell lines [66]. In addition, it has been reported that the down-regulation of HFN1B gene is associated with progression in hepatocarcinoma [67] and poor prognosis in renal [68] and prostate [69] cancers. Considering all the above but also the fact that the association of the HNF1Brs7501939 SNP with OS was driven by a non-diabetogenic (T) allele that does not affect HNF1B mRNA expression, we hypothesize that the effect of this variant to contribute to tumour progression in MM might be mediated by a non-insulin-dependent mechanism. There was a reasonable amount of regulatory data for the HNF1Brs7501939 SNP that supported evidence of the active role of the HNF1B locus. However, whether elevated HNF1B levels lead to tumour transformation and disease progression is not yet understood and functional studies to examine whether HNF1B variants influence cancer prognosis are lacking.

Another interesting finding of this study was the association of the *BCL11A, MADD, PRC1, PROX1, SCL30A8*, *SLC2A2* and *TCF7L2* SNPs with OS. We found an overall association of the *SLC2A2*_rs11920090T_ and *TCF7L2*_rs7903146T_ alleles with better OS whereas the association of the *SLC30A8*_rs13266634T_, *BCL11A*_rs10490072C_*, PRC1*_rs8042680A_ and *PROX1*_rs340874G_ alleles or the *MADD*_rs7944584T/T_ genotype with OS was restricted to male or female genders. Despite the potential interest of the associations observed for these SNPs with OS, only the association of the *SLC30A8*_rs13266634_ SNP with poor OS in men reached significance at experiment-wide significance threshold. This result suggested a key role of the *SLC30A8* locus in the modulation of overall survival. However, given the consistency of the overall or gender-specific associations observed for *BCL11A, MADD, PRC1, PROX1, SLC2A2* and *TCF7L2* SNPs with OS across the populations tested and considering that gender-specific genetic alterations might influence MM survival [48], we suggest that these variants might also exert a modest effect to modulate patient survival.

*SCL30A8* gene encodes a zinc transporter involved in the control of insulin processing and secretion [70]. Although no previous studies have reported a link between this locus and MM, there are evidences that suggest that zinc transporters might contribute to cancinogenesis [71, 72] through a gender-dependent mechanism [73]. The association of the coding *SLC30A8*_rs13266634_ SNP with OS was due to a non-diabetogenic allele suggesting that, rather than modulating glucose homeostasis and insulin secretion [74], the effect attributed to the *SLC30A8* locus on MM survival might be driven by a direct effect of Zinc in biological processes such as DNA and RNA stabilization [75], binding of protooncogenes to DNA [75–77] and the activation of IGF1 [26, 27, 61] or telomerase [78]. The *SLC30A8*_rs13266634C_ allele has been consistently associated with decreased rates of Zinc transport activitiy and reduced intragranular Zinc levels [79]. However, eQTL data on plasma cells from MM patients did not reveal correlation between this variant and *SLC30A8* mRNA levels suggesting that, rather than regulating gene expression, the T allele affect transporter activity in an allele-dose-dependent manner causing increased Zinc concentration and thereby promoting unlimited proliferation of MM cells, disease progression and poor survival. In addition, regulatory data suggest that the *SLC30A8* locus might play a role in survival through the modulation of specific transcription factors implicated in tumour promotion and dissemination.

As for the *HNF1B* and *SLC30A8* SNPs, the association of the *BCL11A*_rs10490072_ and *MADD*_rs7944584_ SNPs with OS was determined by non-diabetogenic alleles. *BCL11A* functions as a myeloid and B-cell proto-oncogen and has been associated with the development of B-cell malignancies [80, 81] whereas *MADD* encodes for a MAP-kinase activating cell domain involved in the control of physiological cell death through TNF- and caspase-dependent apoptosis [82]. In contrast to these associations, the association of the *TCF7L2*_rs7903146,_*SLC2A2*_rs11920090_ *PRC1*_rs8042680_, and *PROX1*_rs340874_ SNPs with OS was driven by diabetogenic alleles, which suggested that the effect of these variants on OS might be explained by their regulatory effect on insulin secretion and, consequently, on cell proliferation and tumour cell growth. Whereas *SLC2A2* encodes a highly efficient glucose transport that is expressed in pancreatic cells and regulates insulin secretion by modulating entry of glucose into the pancreatic cell [83], TCF7L2, PRC1 and PROX1 are proteins that have been involved in β-cell survival and function [84] and in glucose and nonesterified fatty acids or branched-chain amino acids metabolism in liver [85, 86]. Despite these interesting results and the regulatory data observed for all these SNPs, the lack of information regarding T2D status among MM patients did not allow us to ensure that the observed effect of the TCF7L2, SLC2A2, PRC1, and PROX1 SNPs on OS could not be due to a different distribution of diabetic patients when grouping by genotype or gender.

This study had both strengths and limitations. Strengths include the use of relative large discovery and replication populations that allowed us to validate the most interesting associations. Limitations include lack of information regarding the classical genetic prognostic factors (chromosomal abnormalities, etc.), T2D status and a relatively small statistical power to detect modest associations with OS, especially when gender-stratified analysis were performed. Another limitation was the partial overlapping of genetic information between studies and the use of imputed genotypes that did not allow to perform a reliable validation of the association observed for genetic variants within *ADAMTS9, BCL11A, KCNQ1, MADD* and *PROX1* genes with overall survival.

In conclusion, this study reports the first evidence of an association between the *HNF1B*_rs7501939_ SNP and OS for MM and suggests that the *HNF1B* locus might, likely through a non-insulin-dependent mechanism, play an important role in modulating MM prognosis. Likewise, this study shows a strong association of the *SLC30A8*_rs13266634_ SNP with poor OS in men that might, at least in part, account for gender differences in OS. Additional studies using larger and well-characterized populations are needed to further replicate these findings but also those involving the *BCL11A, MADD, PRC1, PROX1,* *SLC2A2* and *TCF7L2* loci on OS.

## MATERIALS AND METHODS

### Patients, clinical data collection and survival endpoint definition

A total of 1420 Caucasian MM patients were ascertained through the IMMEnSE consortium. Full details of this consortium have been published elsewhere [87]. In brief, inclusion criteria were newly diagnosed MM with Salmon & Durie stage I, II and III, age 18–90 years inclusive and Caucasian origin. DNA was purified from blood specimens using the QIAamp DNA Blood Mini Kit (Qiagen) and clinicophathological characteristics including age, gender, country of origin and disease stage (Durie-Salmon) were retrospectively gathered from medical records in each participant institution (Table 1). Diagnosis of patients with symptomatic MM was carried out by hematologists according to the International Myeloma Working Group (IMWG) criteria [88, 89]. All patients within the IMMENSE consortium for whom survival information was available were included in the study (936 MM cases, 454 women and 482 men) (Table 1). All participants gave their written informed consent to participate in the study.

### SNP selection and genotyping

Fifty-eight variants were selected based on the GWAS for T2D [84, 90–126] and were genotyped in the IMMEnSE consortium population (Table 4). We considered only SNPs that were replicated in large and independent populations or which came up in several GWAS or their meta-analyses. Additional criteria were potential functionality and linkage disequilibrium (LD) between the reported SNPs. The genotyping of the selected polymorphisms was carried out at GENYO (Centre for Genomics and Oncological Research: Pfizer/University of Granada/Andalusian Regional Government, Granada, Spain) using KASPar^®^ assays (LGC Genomics, Hoddesdon, UK) according to manufacturer's instructions. For internal quality control, 5% of samples were randomly selected and included as duplicates. Concordance between the original and the duplicate samples for the 58 SNPs was ≥ 99.0%. Call rates for all SNPs were ≥ 90.0% with the exception of the WFS1_rs734312_ SNP that was excluded from further analyses.

**Table 4:** Selected type-2 diabetes-related SNPs

Gene name	dbSNP rs#	Nucleotide substitution	Reference allele IMMENSE	GWAS-identified risk allele for T2D	Location/Aa substitution	References
*ADAM30*	rs2641348	T/C	T	C	L359P	[103, 124]
*ADAMTS9*	rs4607103	**T**/C^1^	C	C	Near gene	[84, 104, 124]
*ADCY5*	rs11708067	T/**C^2^**	T	T	Intronic	[96, 111]
*ADRA2A*	rs10885122	G/T	G	G	Near ADRA2A	[96]
*ARAPI, CENTD2*	rs1552224	G/T	T	T	Near gene	[105, 120]
*BCL11A*	rs10490072	C/T	T	T	Near gene	[124]
*CDC123, CAMK1D*	rs12779790	A/**G^3^**	A	G	Near gene	[84, 104, 124]
*CDKAL1*	rs7754840	C/G	G	C	Intronic	[95, 97, 113]
*CDKN2A-2B*	rs564398	T/**C^4^**	T	T	Near gene	[84, 95, 104, 113, 116, 122, 124]
*CDKN2A-2B*	rs10811661	T/C	T	T	Near gene	
*CDKN2A-2B*	rs2383208	A/G	A	A	Near gene	
*COL5A1*	rs4240702	C/T	C	n/s	Intronic	[91]
*CRY2*	rs11605924	A/C	C	A	Intronic	[96]
*DCD*	rs1153188	A/T	A	A	Near gene	[124]
*EXT2*	rs1113132	C/G	C	C	Intronic	[97, 114]
*FADS1*	rs174550	C/T	T	T	Intronic	[96]
*FAM148B*	rs11071657	A/G	A	A	Near gene	[93, 96]
*FLJ39370*	rs17044137	A/T	T	A	Near gene	[95]
*FTO*	rs8050136	A/C	C	A	Intronic	[104, 125, 126]
*G6PC2*	rs560887	G/A	G	G	Intronic	[91, 92, 94, 96, 107]
*GCK*	rs1799884	G/A	G	A	Near gene	[91, 92, 94, 96, 107]
*GCKR*	rs1260326	C/**T^5^**	C	T	L445P	[91, 96, 111]
*HHEX*	rs1111875	G/A	G	G	Near gene	[95, 97, 104, 113, 114, 125, 126]
*HMGA2*	rs1531343	C/G	G	C	Near gene	[105, 120]
*HNF1A (TCF1)*	rs7957197	A/T	T	T	Intronic	[105, 120]
*HNF1B (TCF2)*	rs7501939	C/**T^6^**	C	C	Intronic	[101, 110]
*IGF1*	rs35767	C/T	C	C	Near gene	[96, 106]
*IGF2BP2*	rs4402960	G/T	G	T	Intronic	[84, 95, 97, 104, 113, 125, 126]
*IL13*	rs20541	C/**T^7^**	C	T	R144Q	[95]
*IRS1*	rs2943641	C/T	C	C	Near gene	[109, 117, 120]
*JAZF1*	rs864745	A/G	A	A	Intronic	[84, 124]
*KCNJ11*	rs5215	T/**C^8^**	T	C	V337I	[95, 98, 104, 113, 121, 125, 126]
*KCNJ11*	rs5219	C/T	C	T	K23E	
*KCNQ1*	rs2237897	C/T	T	C	Intronic	[118, 119, 122, 123]
*KCNQ1*	rs2074196	G/**T^9^**	G	G	Intronic	
*KCNQ1*	rs2237892	C/T	C	C	Intronic	
*KCNQ1*	rs2237895	A/C	A	C	Intronic	
*KCNQ1OT1*	rs231362	G/A	G	G	Intronic	[105, 118, 120]
*LTA*	rs1041981	A/C	A	A	T60N	[102]
*MADD*	rs7944584	A/**T^10^**	A	A	Intronic	[96]
*MCR4*	rs12970134	A/G	G	A	Near gene	[93]
*MTNR1B*	rs1387153	C/T	C	T	Near gene	[91, 107, 120]
*NOTCH2*	rs10923931	G/T	G	T	Intronic	[104, 124]
*PKN2*	rs6698181	C/T	C	T	Intergenic	[95]
*PPARG*	rs1801282	C/**G^11^**	C	C	P12A	[90, 95, 104, 113, 121, 124–126]
*PRC1*	rs8042680	A/C	C	A	Intronic	[105, 120]
*PROX1*	rs340874	A/**G^12^**	A	G	Promoter	[96]
*RBMS1*	rs7593730	C/T	C	T	Intronic	[108]
*SLC2A2*	rs11920090	A/T	A	T	Intronic	[96]
*SLC30A8*	rs13266634	C/**T^13^**	C	C	R325W	[84, 90, 95–97, 104, 113, 114, 125, 126]
*TCF7L2*	rs7903146	C/**T^14^**	C	T	Intronic	[95–97, 99, 104, 11–115, 125, 126
*TCF7L2*	rs12255372	G/**T^15^**	G	T	Intronic	
*THADA*	rs7578597	T/C	T	T	T1187A	[124]
*TP53INP1*	rs896854	A/G	G	G	Intronic	[105, 120]
*TSPAN8*	rs7961581	C/T	T	C	Near gene	[100]
*VEGFA*	rs9472138	C/T	C	T	Near gene	[124]
*WFS1*	rs734312	A/G	A	n/s	H611R	[110]
*WFS1*	rs10010131	A/G	G	G	Intronic	[110]

n/s, not specified; Aa, Aminoacid; GWAS, genome-wide association studies; OS, overall survival.

References are listed in Supplementary Material. Effect allele in bold and underlined.

1 T/T genotype was associated with poor OS in women with an opposite but not significant effect in men.

2 C allele was associated with better OS in women with no effect in men.

3 G allele was associated with better OS in women with no effect in men.

4 C/C genotype was associated with better OS. No gender-specific effect was observed.

5 T/T genotype was associated with poor OS. No gender-specific effect was observed.

6 T allele was associated with poor OS. No gender-specific effect was observed.

7 T/T genotype was associated with poor OS in women with an opposite but not significant effect in men.

8 C allele was associated with poor OS in men with an opposite but not significant effect in women.

9 T allele was associated with poor OS. No gender-specific effect was observed.

10 T/T genotype was associated with better OS in men with no significant effect in women.

11 G allele was associated with better OS in men with an opposite but not significant effect in women.

12 G allele was associated with poor OS in women with an opposite but not significant effect in men.

13 The presence of each additional copy of the T allele was associated with poor OS in men with no effect in women (additive effect).

14 T allele was associated with better OS in men with no effect in women.

15 T allele was associated with better OS in men with an opposite but not significant effect in women.

### Replication

For replication purposes, seven hundred MM patients (296 women and 404 men) were provided by the University Clinic of Heidelberg (Germany). This cohort consists of 98 GMMG-HD3 trial patients, 291 GMMG-HD4 trial patients and 311 patients transplanted in Heidelberg but not enrolled in clinical trials (Table 1). Ethical approval for these patients and written informed consent of trial patients was also obtained. Clinical and survival data were prospectively collected for trial patients on case report forms and retrospectively gathered from medical records for none-trial patients. Genetic information of 53 SNPs (36 genotyped SNPs and 17 imputed SNPs) was extracted from the GWAS conducted in the Heidelberg cohort. After imputation, no information was available for 5 SNPs.

### *In silico* functional analysis

Haploreg (http://www.broadinstitute.org/mammals/haploreg/haploreg.php) and ENCODE annotation data (https://genome.ucsc.edu/ENCODE/) were used to predict the functional role of potentially interesting SNPs.

### eQTL analysis

We also assessed whether selected SNPs correlated with mRNA expression levels in a public eQTL browser for peripheral blood mononuclear cells (http://genenetwork.nl/bloodeqtlbrowser/) [54]. Expression quantitative trait loci (eQTL) data on malignant plasma cells of 658 patients from the University Clinic of Heidelberg (Germany) were also available for this study. Detailed information on sample collection and clinico-pathological characteristics of MM patients as well as technical details of gene expression analysis have been published elsewhere [127].

### Statistical analysis

We used chi-square tests to assess Hardy–Weinberg Equilibrium (HWE) for each SNP among IMMEnSE patients. The primary outcome was OS and the endpoint was defined as death from any cause. Survival time was calculated as the time from MM diagnosis (discovery population) or the first stem cell transplantation (replication population) until the occurrence of the study endpoint, censoring at the date of death or the last observed follow-up time. Association with OS defined as hazard ratio (HR) was calculated for each SNP using Cox regression multivariate analysis adjusted for age, gender, country of origin and Durie-Salmon stage (IMMENSE cohort) or for age, gender and clinical trial (Heidelberg cohort). Association estimates were calculated according to dominant, recessive and log-additive models of inheritance with the major allele as reference for regression analyses (Table 4). We also performed gene-gender interaction analyses to determine whether the association between SNPs and MM OS was of similar magnitude in men and women. Survival function was displayed using the Kaplan-Meier method [128] and survival differences across genotypic groups were analysed using the log-rank test.

In order to account for multiple comparisons, we used the Meff/MeffLi method [129], which calculates the effective number of independent genetic markers analysed (*N* = 54) on the basis of the spectral decomposition (SpD) of matrices of pairwise LD between SNPs (http://neurogenetics.qimrberghofer.edu.au/SNPSpDlite). In addition, we also considered the number of genetic inheritance models tested (dominant, recessive and log-additive). This resulted in a study-wide significance threshold of 0.00031 ([0.05/54]/3) to keep type I error rate at 5%.

Finally, in order to confirm significant associations, a meta-analysis combining genetic data obtained in the IMMENSE population with those extracted from the GWAS conducted in the Heidelberg cohort was also performed following dominant, recessive and additive models of inheritance. The I^2^ statistic was used to assess heterogeneity between both studies and the pooled HR was computed using the random-effect model (assuming that between-study variation might depend on chance or random variation and an individual study effect). Random-effects models are more conservative than fixed-effects models and give rise to wider confidence intervals (CI), which ensures the reliability of the results even though the data come from studies with a relatively different design. All statistics were calculated using SPSS (v.20) and STATA (v.12) for MAC.

## SUPPLEMENTARY TABLES










